# Review of Non-Destructive Testing for Wind Turbine Bolts

**DOI:** 10.3390/s25185726

**Published:** 2025-09-13

**Authors:** Hongyu Sun, Jingqi Dong, Hao Liu, Wenze Shi, Qibo Feng, Kai Yao, Songling Huang, Lisha Peng, Zhichao Cai

**Affiliations:** 1 School of Physical Sciences and Engineering, Beijing Jiaotong University, Beijing 100044, China; 23341053@bjtu.edu.cn (J.D.); 23341115@bjtu.edu.cn (H.L.); qbfeng@bjtu.edu.cn (Q.F.); kaiy@bjtu.edu.cn (K.Y.); 2 Key Laboratory of Nondestructive Testing, Ministry of Education, Nanchang Hangkong University, Nanchang 330063, China; 70658@nchu.edu.cn; 3 State Key Laboratory of Performance Monitoring and Protecting of Rail Transit Infrastructure, East China Jiaotong University, Nanchang 330013, China; zccai@ecjtu.edu.cn; 4 State Key Laboratory of Power System, Department of Electrical Engineering, Tsinghua University, Beijing 100084, China; huangsling@tsinghua.edu.cn (S.H.); penglisha@mail.tsinghua.edu.cn (L.P.)

**Keywords:** nondestructive testing, wind turbine bolts, bolts defects, deep learning

## Abstract

As the world increasingly gravitates towards green, environmentally friendly and low-carbon lifestyles, wind power has become one of the most technologically established renewable energy sources. However, with the continuous increase in their output power and height, wind turbine towers are subjected to higher-intensity alternating wind loads. This makes critical components more prone to fatigue failure, potentially leading to major accidents such as tower buckling or turbine collapse. High-strength bolts play a vital role in supporting towers but are susceptible to fatigue crack initiation under long-term cyclic loading, necessitating regular inspection. Types of wind turbine bolts mainly include high-strength bolts, stainless steel bolts, anchor bolts, titanium alloy bolts, and adjustable bolts. These bolts are distributed across different parts of the turbine and perform distinct functions. Among them, high-strength bolts in the tower are particularly critical for structural support, demanding prioritized periodic inspection. Compared to destructive offline inspection methods requiring bolt disassembly, non-destructive testing (NDT) has emerged as a trend in defect detection technologies. Therefore, this review comprehensively examines various types of NDT techniques for wind turbine towers’ high-strength bolts, including disassembly inspection techniques (magnetic particle inspection, penetration inspection, intelligent torque inspection, etc.) and non-disassembly inspection techniques (ultrasonic inspection, radiographic inspection, infrared thermographic inspection, etc.). For each technique, we analyze the fundamental principles, technical characteristics, and limitations, while emphasizing the interconnections between the methodologies. Finally, we discuss potential future research directions for bolt defect NDT technologies.

## 1. Introduction

With the growing popularity of green and low-carbon lifestyles, countries worldwide increasingly favor clean and efficient energy sources. Against the global backdrop of renewable energy integration and the transition toward a low-carbon future, nations prioritize developing new-generation power systems to achieve “carbon peak and carbon neutrality” goals, ensuring energy security and sustainable development strategies. The types of energy harnessed by these new types of power systems include renewable energies such as solar energy, wind energy, and water energy, and technologies for utilizing renewable energies have developed rapidly since the world energy crisis became part of the public conversation in the 1970s. In the development of renewable energy technology, wind power generation technology, because of its established technology, good infrastructure, and relative cost competitiveness, has the advantage [1]; so wind power generation is the principal objective of new power systems; Both onshore and offshore wind power generation technologies relatively well established, and China’s wind energy resources are abundant; thus wind power generators are playing an important role in achieving China’s “double carbon” goal and low-carbon energy transition [2]. In industrial applications, wind power generation also plays an irreplaceable role; wind turbines are employed in the energy conversion and fluid transmission of nuclear core equipment, as well as in the petrochemical and aerospace sectors, among others. According to the latest report released by the Global Wind Energy Council (GWEC), global wind power capacity reached a record 117 gigawatts (GW) in 2024, and the cumulative installed capacity reached 1136 GW. In Europe, the second largest wind energy market, the share of wind power generation has reached 17%, surpassing natural gas generation and becoming the second major source of electricity generation in the European Union (EU) for the second consecutive year [3].

**Figure 1:**
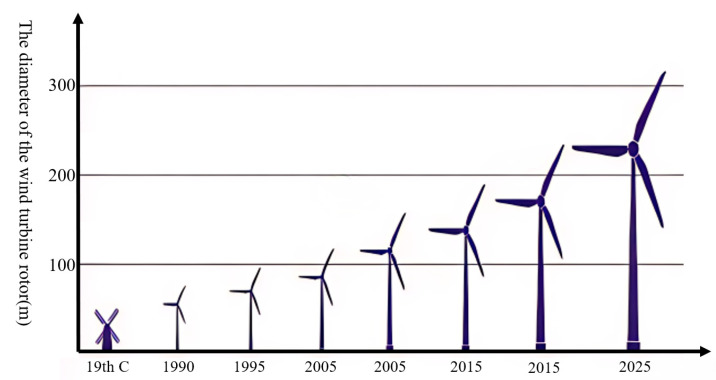
Trend of wind turbine impeller diameter [5]. This figure illustrates that as time progresses, the rotor diameter has been increasing year by year.

**Figure 2:**
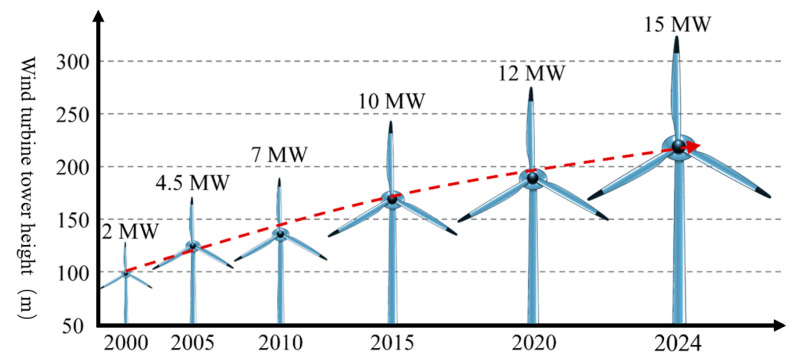
Trend of wind turbine tower height. This figure shows that as time goes by, the height of wind turbine towers has been increasing year by year.

**Figure 3:**
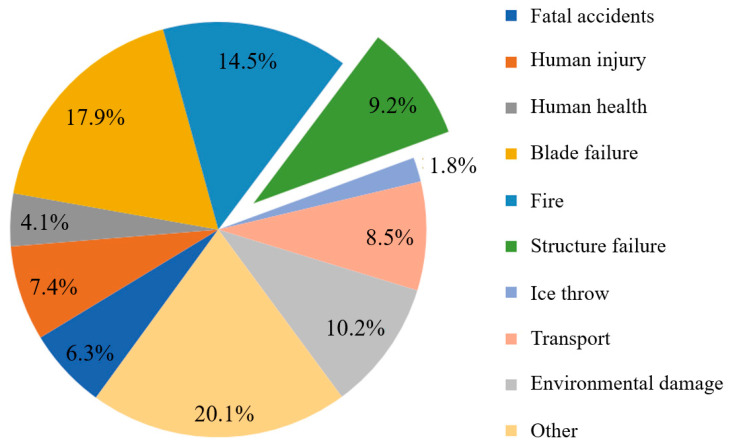
Distribution of failure types of wind turbine accidents from 1980 to 2016 [4].

As the world’s demand for electricity continues to grow, the installed capacity of wind turbines increases, and the diameter of the wind turbines increases accordingly (as shown in Figure 1). Moreover, the height of the tower needs to be increased in order to capture more wind energy, so the height of the wind turbine needs to be continuously increased to meet the increase in installed capacity (as shown in Figure 2). However, due to the long-term operation of wind turbines in harsh environments, along with the increase in the manufacturing height of wind turbines, the tower needs to withstand higher-intensity alternating wind loads, gravity loads, and dynamic loads during operation, which can lead to fatigue failure in critical parts of the structure, and even to major accidents such as the tower bending and the wind turbine collapsing [2]. Wind turbine bolts are crucial fasteners in wind turbines; they ensure reliable connections between the various parts of the structure. Cracks, loose internal defects, and other problems occurring due to the large axial preload, will directly affect the structural stability of the tower, leading to the deformation or displacement of the tower when it is subjected to a high wind load, which will not only cause substantial economic losses but could also threaten the lives and safety of nearby personnel. Over a period of four decades up to 31 December 2016, a total of 1999 wind farm accidents occurred. Structural failures, including tower collapse and turbine damage, accounted for 9.2% of the total accidents (as shown in Figure 3). For example, the collapse of a wind turbine tower in Lemnhult, Sweden, in 2015 was caused by bolt fatigue [4]. Therefore, to ensure the preservation of equipment and the safety of personnel during the service life of a wind turbine, regular, periodic inspection of the fatigue damage of wind turbine bolts is required [1].

In order to better prevent wind turbine collapse accidents due to bolt fatigue, we need to develop a clear understanding of the different types of wind turbine bolts and the different roles they play. The main classifications of wind turbine bolts, as well as the parts they connect and the roles they play, are summarized in Table 1. Turbine bolts are mainly divided into high-strength bolts, stainless steel bolts, anchor bolts, titanium alloy bolts, adjustable bolts, and other bolts. These bolts are situated in different parts of the turbine and each has different functions [5]. High-strength bolts are mainly used in the tower sections, i.e., the nacelle or the tower generator or other parts of the tower, and play a role in connecting the load bearing; stainless steel bolts are used for offshore wind farm tower connections, being corrosion-resistant and known for their superior performance; anchor bolts are used to secure the wind turbine foundation to ensure the turbine’s stability in strong winds and other extreme conditions; titanium alloy bolts are mainly used for rotor blade connections and for other parts that require lightweight design and improved durability; and adjustable bolts are also used for rotor blade connections, as well as for bearing connections and other parts that require the automatic adjustment of the degree of relaxation. High-strength bolts, with their excellent tensile strength and fatigue resistance, coupled with the use of special anti-corrosion coatings and heat treatment processes to ensure that the connections in the tower tube are reliable and adaptable to harsh environments, are evidently the most important bolts used in the structure of a wind turbine. Therefore, the NDT method in this article focuses on the particular defects of high-strength bolts.

In order to more effectively detect defects in the high-strength bolts, we need to develop a clearer understanding of the causes of such defects. The causes of bolt defects are complex, involving a variety of factors such as material manufacturing and the environment [1]. Defects produced in the manufacturing process include quenching cracks and internal inclusions, but bolt defects, including fatigue cracks, stress corrosion cracks, thread wear, hydrogen delayed fracture inclusions, porosity, etc., are more often caused by harsh environments. These defects are a potential threat to the safe operation of the turbine and must be inspected regularly. However, traditional bolt detection methods, such as disassembling the high-strength bolts of the wind turbine tower for destructive metallographic analysis, cannot meet the needs of modern wind turbines with long-term operation. Non-destructive testing technologies, such as ultrasonic detection rays, electromagnetic detection, etc., can perform the real-time monitoring and online diagnosis of bolt defects. Non-destructive testing technologies are not only applied in bolt defect detection, but also in manufacturing; to detect defects such as porosity, inclusions, and cracks in recently parts, they are also used in the aerospace industry to screen for defects in the aircraft fuselage, wing engine, and other key parts of the airplane, as well as in the petrochemical industry, the electric power industry, and other fields. Hence, it is clear that non-destructive testing technology represents a major step forward in the field of defect detection.

Industrial applications, whether in the field of wind power generation or in the aerospace, petrochemical, or other energy generation industries, cannot be separated from the detection of defects and fatigue damage. Defect detection requirements and challenges can be described as follows.

(1) High accuracy: Bolt defect detection needs to be accurate to the sub-millimeter or micron level to ensure the safety and stable operation of the equipment.
(2) High resolution: It is necessary to clearly distinguish the shape, size, and location of small defects to help engineers in their subsequent assessments and repairs.
(3) High sensitivity: It is necessary to capture the weak signals in the detection process and to suppress the noise to prevent the further development of defects.
(4) High efficiency: In the mass production or equipment operation, a large number of inspection tasks must be undertaken in a short period of time to meet production schedule and equipment maintenance requirements.
(5) Real-time monitoring: Continuous monitoring of the equipment must be carried out in real time to detect defects in a timely manner and achieve dynamic management.
(6) Defect diversity: Faced with various types of defects, including cracks, holes, corrosion, and wear, detection methods must be able to cover the full range of defects.
(7) Environmental factors: Due to the complexity of the environmental conditions, equipment needs to have a good environmental adaptability and thus must undergo high-temperature and high-pressure testing.

From the perspective of defect detection, detection accuracy and sensitivity are the two most critical indicators, though resolution, efficiency, real-time monitoring, defect diversity, and the impact of external environmental changes should also be considered. With the rapid development of wind turbines, subtle defects will directly affect the operation safety and efficiency of the turbines. Defect detection must therefore be extremely accurate and high-resolution in order to clearly identify the morphology and location of the defects. Since wind turbines often operate in complex and changing environments [1], defects may be very small, diverse, and difficult to detect, so the defect detection system needs to be highly sensitive to weak signal changes. At the same time, as the scale of the wind turbines continues to expand, the efficiency of the defect detection method and our ability to monitor the various workpieces in real time have become important reference factors.

**Figure 4:**
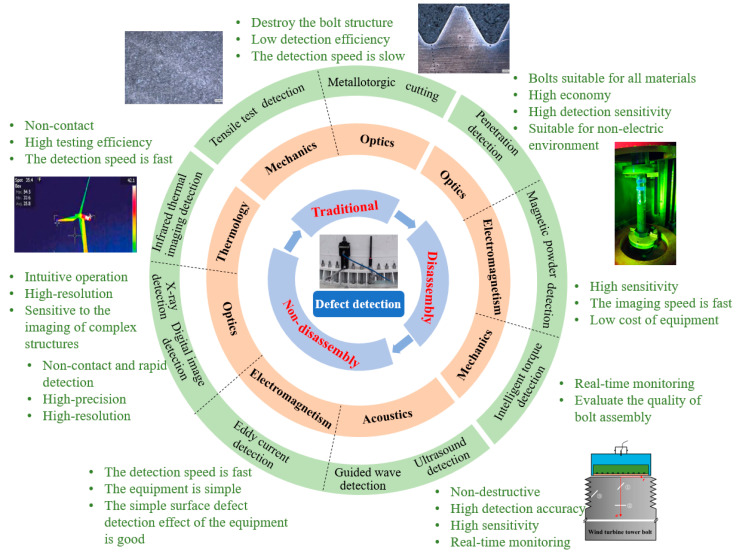
Overview of wind turbine tower bolt defect detection technology [2].

In order to meet these requirements, defect detection technology for wind turbines is constantly being developed and improved. This article presents a comprehensive discussion of the non-destructive testing methods for wind turbine tower bolt defects, their advantages and disadvantages, as well as the particular defects for which each method is employed to detect. An overview of the wind turbine bolt detection technologies can be found in Figure 4. Traditional inspection methods, such as destructive metallographic analysis methods and tensile test inspection methods, are completely destructive to the bolt, so these methods are often used for failure analysis or quality assessment of a bolt, as shown in Table 2. Non-destructive testing technologies have since become the standard approach to wind turbine tower bolt testing; compared to traditional bolt testing methods, non-destructive methods can not only reduce the economic cost and improve the detection efficiency of a single bolt; they can also be used to visualize bolts defects and, most importantly, can meet the accuracy and sensitivity needs of defect detection in industrial applications. Moreover, compared with methods involving the disassembling of the bolt, non-disassembly, non-destructive testing technologies, such as ultrasonic detection, eddy current detection, ray detection, etc., are more in line with the demand for large-scale, in-service wind turbine bolts in the process of wind turbine development. Furthermore, these methods meet the requirements of high accuracy, high sensitivity, and high resolution; i.e., not only do they not damage the structure of the bolt, they also accurately detect defects inside the bolt, determining the size, shape, and location of defects to provide a reliable basis for improving defect detection efficiency. This paper reviews the non-destructive testing methods for detecting wind turbine tower bolts, including disassembly, non-destructive testing technology and non-disassembly, non-destructive testing technology, with the former including magnetic particle testing, penetration testing, and intelligent torque testing, and the latter including ultrasonic testing, guided wave testing, eddy current testing, fiber-optic grating sensing detection, digital image detection, ray detection, and infrared thermal imaging detection. We cover the principles of measurement, the histories of each technology, their applications, their advantages and disadvantages, and future development directions.

Although there have been literature reviews on structural health monitoring or bolted connection technologies for wind turbine systems, systematic reviews specifically focused on non-destructive testing (NDT) techniques for high-strength bolts in wind turbines remain relatively scarce. Building upon existing research, this paper presents, for the first time, a comprehensive comparative and integrative analysis of both disassembly-based and in situ NDT techniques, with particular emphasis on the latest applications and advances of emerging technologies—such as ultrasonic phased arrays, guided waves, eddy current, infrared thermography, and digital image detection—in the inspection of wind turbine bolts. Furthermore, this review systematically summarizes the physical principles, applicable scenarios, and technical limitations of various methods, and incorporates, also for the first time, the application prospects of artificial intelligence and deep learning in bolt defect recognition and predictive maintenance. Thereby, it fills a gap in the existing literature regarding technology integration and intelligent development trends, providing more comprehensive technical reference and future directions for both scientific research and engineering practice in this field.

## 2. Disassembly Inspection Techniques

This section primarily presents an overview of the development history, fundamental principles, advantages, and disadvantages of the inspection technologies for wind turbine tower flange bolts during disassembly. It also analyzes which types of defects each technology (including magnetic particle testing, penetrant testing, and intelligent torque inspection) is better suited to. Finally, a summary of the above content is provided [6].

This section will provide a detailed introduction to Magnetic Particle Inspection (MPI) and Dye Penetrant Inspection (PT). They are not only the most classic but also the most widely used two major techniques for surface inspection. Furthermore, in the specific context of wind turbine bolts, which are mostly made of ferromagnetic materials (high-strength steel), the applicability and importance of MPI far exceed those of PT (PT is more suitable for non-ferromagnetic materials like stainless steel or titanium alloys). Therefore, PT will be presented as a complement to MPI, with a brief introduction to its principles and limitations, while substantial coverage will be devoted to the more targeted MPI.

### 2.1. Magnetic Particle Inspection

**Figure 5:**
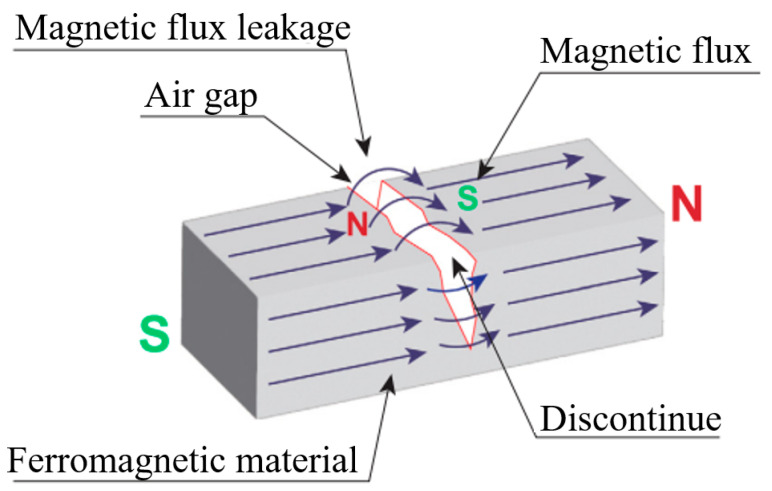
Schematic diagram of magnetic particle inspection: formation of magnetic leakage [8].

Magnetic particle inspection (MPI) is one of the oldest and most established non-destructive testing techniques [7]. It is widely used in the detection of surface and near-surface defects of ferromagnetic components in vehicle engineering, heavy aerospace industry, and other industries based on the that when a strong magnetic field is applied to the surface of a ferromagnetic material or component, the geometrical discontinuity of the surface of the object under inspection will cause the applied magnetic field to leak out from the discontinuity, forming a magnetic leakage or a distortion of the magnetic lines of force, as shown in Figure 5 [8]. Magnetic particles are introduced to the surface of the inspected object in this condition, which are attracted to the leakage field and adhere to the defects, making them observable. Magnetic leakage signals can be recorded and analyzed to invert the characteristic parameters of the defects and thus assess the defect size [8]. Also, the width (W) and height (H) of the particles attracted by the magnetic leakage and adhering to the defects can be quantitatively determined, and the range of crack widths can be estimated by the linear increase in the aspect ratio of the magnetic particles (W/H) and the depth of cracks, as shown in the study of Fukuoka K. et al. [9].

**Figure 6:**
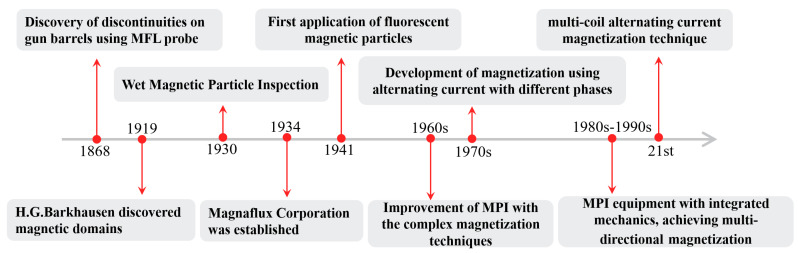
Historical development of magnetic particle inspection [8].

Magnetic particle inspection (MPI) technology has gone through several important stages of development since the discovery of magnetic domains by H. G. Barkhausen in 1868, as can be seen in Figure 6 below. The first flux leakage probe was used to detect discontinuities in gun barrels in 1919; the technique was applied to solve cracking problems in oil well drill pipes in 1928; in 1938, the addition of wetting agents and preservatives to water-based magnetic levitation was patented; multi-directional magnetization was achieved in the 1980s–1990s; and multi-coil AC magnetization was developed in the 21st century. These advances demonstrate the importance and continuous progress of MPI technology in industrial inspection [10,11,12].

Magnetic particle inspection (MPI) technology has been widely used in various industries. MPI technology can detect more than ferromagnetic materials; it can also be used to detect materials such as steel and nickel-based alloys, and it can be applied to workpieces of various shapes and sizes, achieving very high precision, i.e., detecting cracks and scratches at the micron level. Furthermore, MPI can show the defect phenomenon visually through the aggregation of distinctly colored or fluorescent magnetic particles, which is convenient and easy to use. It straightforwardly reveals defect phenomena and allows engineers to quickly assess the defects; hence, it is widely used in aerospace, track defect detection, and other fields. However, such intuitive estimation does not take into account the detailed evaluation of the specific sizes and characteristics of defects by magnetic particle inspection. In reality, defects are usually complex and need to be characterized by a combination of multiple parameters, such as the width of the defect, the thickness and shape of the defect type, or the edges of some special defects, which significantly affect the resulting magnetism leakage, which is based on the leakage signals recorded by sensors according to the Leakage Signal Analysis Model (LSAM). The inverse performance of the many characterizing defect parameters to derive a clear defect signature is obviously difficult, and a lot of effort needs to be devoted to the development of an analytical model with which to interpret and analyze the leakage signals [13,14,15,16,17,18,19,20,21], which also significantly increases the technical cost and the technical requirements for engineers. It has also been suggested that the treatment of the elastic–plastic zone near the crack remains a challenge because the effect of plastic deformation on the magnetic properties of ferromagnetic materials is not yet thoroughly understood [22], which also adds a number of constraints on the surfaces and configurations of the component being inspected.

### 2.2. Penetration Testing

**Figure 7:**
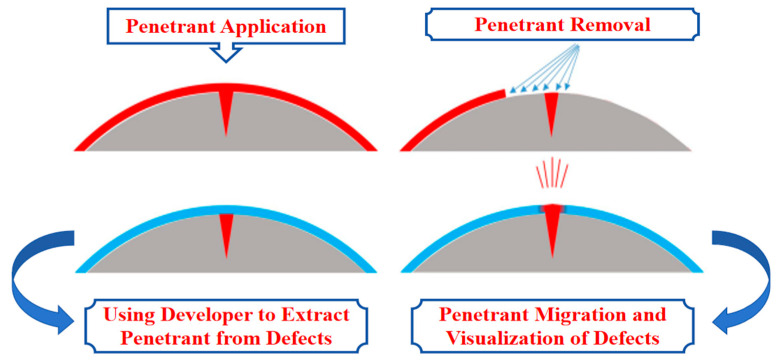
Dye penetrant inspection method comprising the following steps: 1. applying a penetrant to a defect on a surface; 2. removing excess penetrant from the surface; 3. applying a developer to draw the penetrant out of the defect; and 4. diffusing the penetrant around a rupture opening in the surface and visualizing the defect [28].

**Figure 8:**
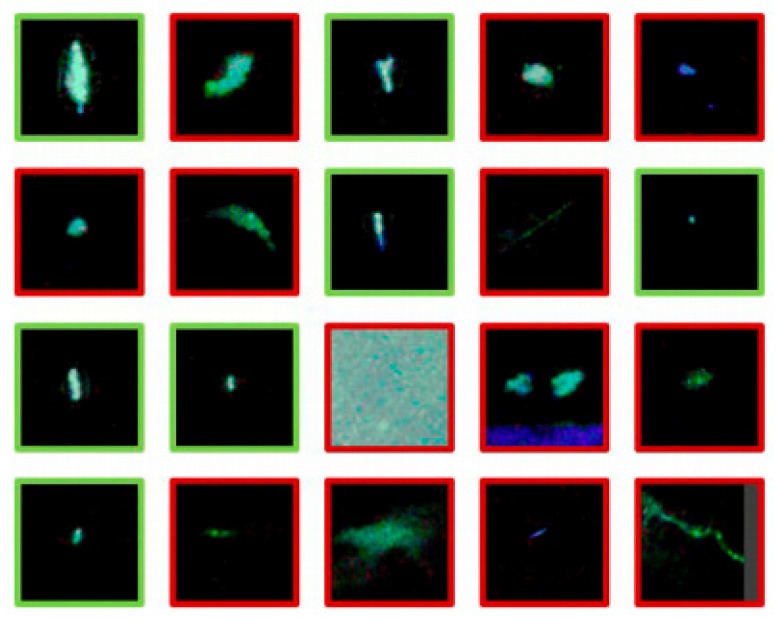
Examples of defects (green border) and error indications (red border) [26].

Penetration testing (PT) is a highly sensitive and widely used non-destructive testing method, commonly used to detect surface damage defects of the component to be tested, as shown in Figure 7. The basic principle is that PT is carried out by adding a low-viscosity penetrant (dye or fluorescent liquid) to the surface of the component to be tested such that the low-surface-tension fluid penetrates the geometrical discontinuities or defects on the surface of the object via capillary action [23]. After the penetration, the excess penetrant is removed from the surface, and a developer is applied. The developer will extract the penetrant from the defect during the development period, creating an observable inspection indication that reveals the location and shape of the defect [24]. Depending on the type of penetrant used, penetrant testing is categorized into two main forms: 1. Visual penetrant testing (VPT), which uses a visible dye and is usually observed under natural or white light. This method is suitable for detecting larger or more obvious defects [25]. 2. Fluorescent penetrant testing (FPT), which uses fluorescent dyes. This method involves placing the part under ultraviolet light to allow engineers to systematically inspect the part and assess the impact of defects through observation, as shown in Figure 8. Fluorescent penetrant testing has a much higher sensitivity and is able to detect smaller defects [23,26,27].

The history of penetrant inspection dates back to the late 1800s when paraffin and oil-based dyes were used to detect cracks on metal surfaces [26]. With the development of industrial technology, penetrant inspection techniques gradually improved and were standardized. With the rise of the aerospace industry at the beginning of the 20th century, the requirements for the inspection of materials and components became more demanding, and penetrant inspection techniques were more widely used and improved. The emergence of the fluorescent penetrant in the middle of the 20th century greatly increased the sensitivity of the inspection. Since then, with the advancement of chemical technology, the performance of penetrants has been optimized, and detection methods have become more diversified. With the development of computer vision and artificial intelligence technology in recent years, automated penetrant detection systems have gradually become a research hotspot [29].

Penetrant detection has been widely used in many fields due to its several advantages, such as easy operation, low cost, and its wide range of applications, but at the same time, there are some limitations. In the aerospace field, fluorescence penetration testing (FPT) is widely used to detect surface defects in key components such as aircraft engine blades and fuselage structural components; it can quickly detect small surface cracks, but the subjective experience of the operator is required to be higher, and the results of the visual inspection are easily affected by ambient lighting conditions [23]. In automotive manufacturing, penetration testing is used to detect surface defects in engine blocks, crankshafts, connecting rods, and other components. It is easy to operate and has little cost, but the detection depth is limited; only surface and near-surface defects can be detected. In the field of machining, penetration testing is commonly used to detect surface and near-surface defects in castings, forgings, etc. The scope of application is wide, but the porous materials or materials with high surface roughness have a deleterious effect, and the ability of this technique to quantitatively analyze the defects is limited [26]. In summary, PT is widely used in various industries as a means of non-destructive testing. However, at the same time, there is an urgent need to make greater breakthroughs in automation and intelligence and the quantitative detection of defect characteristics in order to provide a more reliable and accurate means of detection.

### 2.3. Intelligent Torque Inspection

Torque inspection is of great significance in industry, transport, and aerospace, especially in scenarios involving bolted connections, mechanical assembly, and structural health monitoring. Traditional torque detection methods rely on manual operation or simple mechanical devices, which have low efficiency and poor accuracy, and are difficult to monitor in real time. With the development of smart material sensor technology and signal processing technology, intelligent torque detection technology has emerged, providing a new way to solve these problems.

Strain sensors are a widely adopted sensor type in intelligent torque detection. By pasting strain gauges on the surface of the object to be measured, the change in strain produced when the object is subjected to force can be measured; thus, the torque can be calculated indirectly. For example, in bolting, strain gauges can be mounted onto bolts or nuts, and the preload or torque change in the bolt can be deduced by measured strain responses [30]. Acoustic methods use the propagation characteristics of sound waves in materials to detect torque. For example, the propagation speed and attenuation of ultrasonic waves in a material are affected by the state of stress, and the torque can be deduced by measuring the propagation time or frequency change in the ultra-acoustic waves [31]. In addition, distributed acoustic sensing (DAS) technology can also be used to monitor the loosening of railway track bolts in real time [32]. Electrical methods mainly utilize the properties of piezoelectric materials. Piezoelectric materials produce charge or voltage changes when subjected to stress, and torque detection can be achieved by measuring these electrical signals. For example, piezoelectric ceramics can be mounted as sensors in bolts or structures for the real-time monitoring of torque changes [30].

Early torque detection relied heavily on mechanical torque spanners and torque transducers. Although these tools can provide some torque measurement function, there are problems such as low accuracy and complex operation, which is difficult to monitor in real time [33]. With the development of smart materials (such as piezoelectric materials, shape memory alloys, etc.) at the end of the twentieth century, the torque detection technology began to develop in the direction of intelligence. These materials are able to convert mechanical energy into electrical or thermal energy, which provides a new means for torque detection. The rapid development of sensor technology in recent years has made torque detection more accurate and efficient. At the same time, the application of signal processing technologies (such as machine learning, deep learning, etc.) makes the torque detection system capable of automatically identifying and analyzing complex signals, which further improves the accuracy and reliability of detection.

Intelligent torque detection technology has a wide range of applications in several fields due to its high accuracy, real-time monitoring, and automation features that make it an indispensable tool in modern industry and engineering. In industrial manufacturing, this technology can be used to monitor the preload of bolts in real time to ensure the quality of assembly and avoid safety hazards caused by loose or over-tightened bolts. In the aerospace field, intelligent torque detection technology is used to monitor the bolt connection status in the structure of the aircraft, to detect loose or damaged bolts in time, and to ensure flight safety [30]. In railway transport, distributed acoustic sensing technology can monitor the loosening of railway track bolts in real time, providing important data support for railway maintenance [31]. In addition, in smart structures (such as smart bridges, smart buildings, etc.), smart torque detection technology can be used to monitor the stress distribution and connection status in the structure, providing support for structural health monitoring [32]. However, there are some drawbacks to smart torque detection techniques, such as high system development and deployment costs, especially in large-scale applications, and the fact that some techniques (e.g., acoustic-based detection) may be interfered with by ambient noise, which affects the detection accuracy. In addition, the installation and maintenance of the system is complex and requires specialized technicians to operate.

### 2.4. Summary

Three inspection techniques based on different physical principles (electromagnetism and optomechanics) have been discussed above, with each suitable for different application scenarios and material properties. Magnetic particle inspection (MPI) uses a strong magnetic field to cause defects on the surface of ferromagnetic materials to generate a leakage phenomenon, which indicates the location of the defects through the aggregation of magnetic particles. Widely used in aerospace, track defect detection, and other fields, it is capable of detecting cracks and scratches at the micron level. Magnetic particle inspection technology is sensitive and intuitive, but struggles to accurately assess defect characteristics, with high technology costs and technical requirements for engineers. Detection by penetration penetrates surface defects with a low-viscosity penetrant, which is then drawn out with a developer to form an observable indication. This method is suitable for detecting surface and near-surface defects, such as in aircraft engine blades and automotive parts. Penetrant inspection is easy to operate and inexpensive, but because it requires subjective experience to assess the observable indication, it requires high operator experience and has a limited depth of detection, which is not effective for porous materials. Intelligent torque inspection utilizes strain sensors, acoustic methods, or electrical methods to measure torque changes, reflecting deformation or defects in the component undergoing testing. It is widely used in industrial manufacturing, aerospace, railway transport, and other fields to monitor the state of bolted connections. Intelligent torque detection has the characteristics of high accuracy, real-time monitoring, and automation, but the development cost is high, and it may be subject to environmental noise interference, installation, and maintenance complexity. These non-destructive testing techniques are important in modern industry, but further development is needed to improve the automation, intelligence, and quantitative analysis of the test.

## 3. Non-Disassembly In Situ Inspection Techniques

This section primarily presents an overview of the development history, fundamental principles, advantages, and disadvantages of inspection technologies for wind turbine tower flange bolts during disassembly. It also determines which types of defects each technology (including magnetic particle testing, penetrant testing, and intelligent torque inspection) is better suited to identifying. Finally, a summary of the above content is provided.

This section will elaborate in detail on Ultrasonic Testing (UT) and Radiographic Testing (RT). They are the gold standards for detecting internal defects, with mature technical systems and a profound theoretical foundation. They are also the decisive means for assessing critical internal defects in bolts, such as cracks, pores, and inclusions. Consequently, providing an in-depth explanation (e.g., the phased array technology in UT) is necessary, as it establishes a solid technical foundation for the entire paper.

### 3.1. Ultrasonic Testing

**Figure 9:**
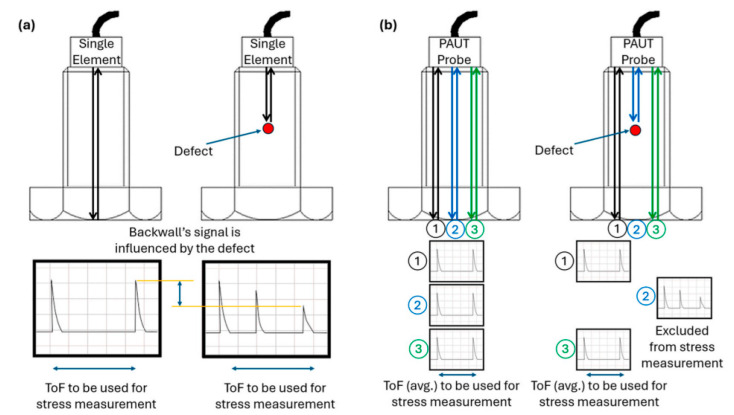
(**a**) Single crystal inspection. (**b**) Phased array inspection [10]. The picture compares the differences in the detection processes between single-crystal detection and phased-array detection.

Ultrasonic testing involves the use of ultrasound in the material propagation of reflection and refraction characteristics to achieve non-destructive, non-disassembly testing, obtaining an acoustic signal containing defect information to determine the presence or absence of defects and their severity. This detection method uses the piezoelectric effect of the material by loading a specific voltage to generate ultrasonic waves. The propagation of ultrasound waves encountering defects will be reflected or refracted; when these reflected or refracted waves propagate again, the piezoelectric material will vibrate at a certain frequency, thus generating a certain voltage signal, which will finally be amplified through the ultrasonic detector and processed and displayed (e.g., Figure 9a). Shah J. K. et al. [34] analyzed the ultrasonic propagation characteristics and interactions at the intersection of the crack and the thread root. Dong M. S. et al. [35] showed that ultrasonic detection methods can effectively detect and quantify cracks as wide as 0.5 mm.

With the increase in the complexity of defects, the existing ultrasonic detection technology is not sufficient to support the industrial development of high-sensitivity, high-precision defect detection. There is an urgent need for the new ultrasonic detection method proposed, namely, ultrasonic phased array non-destructive testing technology. The characterization of defects has always been the technical difficulty in ultrasonic testing, especially for traditional ultrasonic testing technology. Because the traditional ultrasonic detection technology can only display an A-scan waveform, the detector receives too little information; thus, determining the type of defect from the very limited waveform information is very difficult, and most of the ultrasonic detection standards for defect characterization are not met. However, ultrasonic phased array technology is able to obtain information from multiple ultrasonic echoes at the same time, so the phased array probe can image the defect surroundings by considering only the elements that have not encountered the defect [36] (as shown in Figure 9). Chen J. Z. et al. [37] investigated ultrasonic phased array inspection methods for bolts and determined that an acoustic-beam, high-accessibility detection method for internal cracks in complex components is more suitable for the high-precision, online detection and imaging of fatigue cracks in bolts in wind turbine towers with complex structures. Phased array probes have many advantages over the single-crystal probes used in traditional ultrasonic testing. Ultrasonic stress detection relies on the theory of acoustic elasticity, which was first fully described mathematically by Murnaghan, F. (1937) [38], and Javadi, Y. et al. [39] have further shown that ultrasonic phased array detection methods can allow for simultaneous defect and stress detection. It can be seen from the above that we can set up different phased array element structures, combined with certain algorithms, to achieve more complex bolt defect imaging. Holmes, C. et al. [40] have shown that ultrasonic phased array detection is more suitable for the detection and imaging of fatigue cracks in complex structures. The authors proposed an algorithm to improve the defect imaging level by post-processing the data from a static linear ultrasonic array to achieve fast and accurate scanning and imaging of the part to be tested. Kumar, A. [41] achieved the wide-range, accurate-capture, high-resolution imaging of horizontal and vertical groove crack diffraction signals in welded joints. In summary, ultrasonic phased array-focused imaging technology has gained wide attention and achieved relatively promising results in the research literature. This technology is expected to provide a feasible solution for the inspection and imaging of wind turbine tower bolts.

Guided wave inspection is also an important non-disassembly, non-destructive testing technology, the essence of which is to use the guided wave pattern (such as the longitudinal wave, the transverse wave, or the Lamb wave) generated by ultrasonic wave propagation in the medium structure with boundaries (such as bolts, pipeline plates, etc.) for detection, and the defects encountered during the propagation inside the bolts will produce signal changes such as reflections or scattering, and the amplitude, phase, and time delay of the reflected signals will be determined by analyzing the amplitude and phase delay of the reflected signal to determine the presence or absence of defects, the location information, and the severity of defects, as well as to achieve long-distance and large-scale defect detection. Guided wave inspection is divided into axial guided wave inspection and circumferential guided wave inspection. Axial guided wave inspection is more suitable for detecting axial defects in bolts, and circumferential guided wave inspection is more suitable for detecting circumferential defects. The core principle of guided wave detection is the same as ultrasonic detection, which is to judge the defects by transmitting ultrasonic waves and interpreting the echo. Therefore, guided wave detection should also be understood as a kind of ultrasonic detection. However, the core of the technology lies in the waveguide effect through the structural geometry, limiting the wave propagation path such that the energy is concentrated in a specific mode, thus deviating from the traditional ultrasonic detection of distance limitations.

### 3.2. Eddy Current Detection

With the large scale and complexity of wind turbines, the rapid detection of defects on and near the surface of bolts is becoming increasingly urgent. Eddy current inspection, as an efficient non-destructive testing technology, uses electromagnetic induction to identify defects in conductive materials, and the online inspection of wind turbine bolts can be realized without physical contact, which has an important application value in the field of wind power. The essence of eddy current detection is to use the principle of electromagnetic induction; by applying high-frequency excitation externally to induce eddy currents in the conductor, defects will disturb the distribution of the eddy current electric field; then, we measure the changes in the secondary magnetic field so as to detect defects on the surface of the conductive material and near the surface. This detection method is characterized by a fast detection speed and high sensitivity and is commonly used to detect surface cracks and corrosion in the metal parts of turbines.

First, we introduce the development history of eddy current detection: in 1831, Faraday proposed the law of electromagnetic induction; in 1879, Hughes produced the first eddy current used for metal sorting; between 1921 and 1935, the eddy current flaw detector and thickness gauge were invented; in terms of technological innovation, pulsed eddy current (PEC) and array eddy current (AEC) technologies were then developed to improve the detection efficiency and resolution of the method in 2010s, to be later combined with machine learning algorithms to achieve the automatic classification and quantification of defects.

(1) $$\nabla \times \left(\frac{1}{\mu }\nabla \times A\right)+j\omega \sigma A=J_{s}$$

where ▽ is the Hamiltonian operator; *μ* is the magnetic permeability, a physical quantity that describes the magnetic properties of a material and indicates the ability of the material to respond to a magnetic field; ***A*** denotes the vector magnetic potential, a vector field used to describe a magnetic field; *j* denotes the imaginary unit; *ω* denotes the angular frequency, a physical quantity used to describe how quickly or slowly a periodically varying physical quantity can change; σ denotes the electrical conductivity, which describes the electrical conductivity of a material; and ***J_s_*** denotes the vector of source current densities, indicating the distribution of source electrical currents per unit volume. Also included are the distribution of the source electrical currents per unit volume and the source current density vector, reflecting the characteristics of the current source that generates the magnetic field.

In addition to the development of the basic theory of eddy current detection mentioned above, industry is also advancing with the times. See, for example, García-Martín J. et al.’s [42] systematic review of the evolution path of eddy current technology in industrial detection. Further, Huang, L. et al. [43] proposed a quantitative model for wind power bolt crack depth, in which the error is <10%. Wang, T. et al. [44] realized corroded region AI automatic segmentation, with an accuracy of 98.2%. Ribeiro, A. L. et al. [45] developed weather-resistant embedded eddy current sensors for long-term monitoring.

Eddy current detection has become the first choice for screening surface defects on wind turbine metal parts on account of its high efficiency and high sensitivity, but it needs to be complemented with guided wave detection (internal defects) and structured light (deformation quantification) technologies to build a multi-level health-monitoring system.

### 3.3. Radiographic Inspection

With the rapid development of wind power generation technology, the inspection of wind turbine bolts has become an important part of ensuring the safe operation of wind turbines. As a more established non-destructive testing technology, radiographic testing (RT) is widely used in the inspection of wind turbine bolts because it can effectively detect internal defects in the bolts without causing damage to their parts. The principle underlying ray detection technology is that when the ray penetrates the bolt, due to the structure of the bolt, the raw material’s shape and thickness, and other bolt parameters, the ray will vary in terms of its degree of absorption and scattering. Using detectors to determine the intensity of the projection through the bolt, one can determine the internal structure of the bolt; i.e., by analyzing the imaging differences, the different transmission intensities, the density of the bolt, and other parameters, one can identify various abnormalities, such as porosity and cracks [46].

The commonly used radiation sources for radiographic inspection include X-rays and gamma rays. X-rays are generated by a high-energy electron beam impinging on a metal target, and their energy and wavelength can be controlled by adjusting the accelerating voltage and the target material. Gamma rays are produced by the natural decay of radioactive isotopes with a fixed energy and wavelength, such as the decay of cobalt-60 or iridium-192. The main difference between X-rays and gamma rays lies in their energy ranges and penetration capabilities, with X-rays typically being used for inspecting thinner materials or smaller workpieces, whereas gamma rays are more suited to the inspection of thicker materials or larger workpieces [46].

The development of radiographic inspection techniques can be traced back to the beginning of the 20th century, when X-rays were discovered, providing a new means of non-destructive testing. Early radiographic inspection relied heavily on film imaging techniques, whereby images were recorded by placing the film on the opposite side of the workpiece to be inspected and interpreting the change in the intensity of the rays as they penetrated the workpiece. Although this method can detect defects within the material, there are still limitations, such as the long film processing time, low image contrast, and difficulties faced in digitally processing the images [46]. With the development of electronic technology, digital radiography is gradually developing in the direction of digitalization; the technology uses a digital detector to receive ray signals and computer processing to obtain a digital image of the tested part. Digital ray detection technology has an accelerated imaging throughput, high image resolution, and can be digitally stored and processed, alongside several other advantages, greatly improving detection efficiency and accuracy. In recent years, with the introduction of computed tomography (CT) technology, a major breakthrough has been made in volumetric representations with isotropic voxel resolutions. CT technology, through ray scanning from multiple angles, combined with computer algorithms to rebuild the three-dimensional image of the workpiece, can be more intuitive in revealing material and structural defects [46].

Radiographic inspection technology has significant advantages in the inspection of wind turbine bolts. It is capable of detecting small defects within the bolt, such as porosity and cracks, without causing damage to the bolt during inspection, and it is suitable for multiple inspections to monitor changes during use [47]. In addition, radiographic inspection provides high-resolution images that help to accurately identify the location and size of the defects, providing a reliable basis for assessing the quality of bolts [48].

With the continuous progress of technology, the application of radiographic inspection technology in the inspection of wind turbine bolts will see wider use. Future research directions may include the development of more efficient ray inspection equipment, the reduction of inspection costs to improve the degree of inspection automation, and the introduction of artificial intelligence technology to more accurately analyze the inspection results [48]. For example, by introducing machine learning algorithms, defects inside bolts can be automatically identified and classified to improve inspection efficiency and accuracy.

### 3.4. Infrared Thermal Imaging Inspection

**Figure 10:**
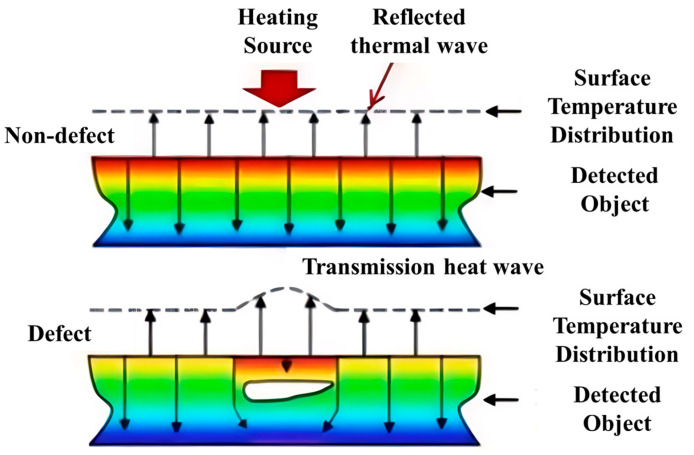
Infrared thermal wave imaging detection principle [52].

Thermal imaging methods are based on the principle of locating defective areas by actively heating the bolt or detecting its natural thermal field using an infrared camera to observe differences in temperature distribution, as shown in Figure 10. It is used in underground defect or anomaly detection. The temperature difference is associated with a difference in thermal diffusivity compared to the ultrasonic portion, thus indicating material irregularities or damage. Thermal imaging methods can be categorized according to the thermal excitation of the subject using passive or active methods. Passive thermography methods are used to study materials at temperatures that are different to (usually higher than) the ambient temperature [49]. Passive methods are not common in wind turbine structural health monitoring and will require more modifications before they show any promise [50]. Active methods use an external stimulus source, such as an optical flash heat lamp, a hot air gun, or a cold air gun, to produce the relevant thermal contrast [14].

Infrared thermography methods (IRT), as more established non-destructive testing techniques, have been widely used in the inspection and stability assessment of materials such as composites, building structural materials artifacts, and plastics [51]. Thermography methods (IRT) can be applied in the detection of defects in metal parts, such as wind turbine tower bolts. The underlying goal is to obtain the radiation flux or temperature change in the bolt through active excitation of the bolt or detection of its natural thermal field; then, to form an image to obtain the defective characteristics of the bolt by analyzing the abnormal change in radiation flux or temperature in the image. The radiation flux or temperature change results from the heat field obtained through both active and passive means [51]. Active excitation is achieved by applying a heat signal to the bolt from a heat source to stimulate the heat field. For undamaged bolt parts, when the heat signal is applied to the bolt, the heat signal will be uniformly propagated inside the bolt, and the radiation flux or temperature change obtained on the surface will also be uniform [52]; however, when there are defects or cracks in the bolt interior or on its surface, the propagation of the heat signal will be reflected, resulting in sudden changes in the radiation flux or temperature changes at the surface of the bolt, as shown in Figure 1. These abnormal, sudden changes in the data illustrate the defective characteristics of the bolt. Passive thermography methods are used to study materials that are different to (usually higher than) the ambient temperature. Passive methods are not common in wind turbine structural health monitoring and will require more modification before they become promising.

The early applications of infrared thermography at the beginning of the 20th century were focused on its military potential. For example, infrared detection technology was used for night reconnaissance and target identification. The technology in this period was mainly based on simple infrared detectors with limited imaging capability. With the development of electronic and computer technology, infrared thermal imaging technology started to progress towards maturity. Infrared thermal imaging technology began to be applied in the industrial field—for example, in the detection of overheating parts in electrical equipment. In the 1980s, the application scope of infrared thermal imaging technology rapidly expanded, covering medical, construction, aerospace, and other fields. In recent years, with the rapid development of sensor technology, image processing technology, and artificial intelligence technology, infrared thermography has entered a new stage. Modern infrared thermal imagers not only have higher resolution and sensitivity but are also able to enhance the accuracy and reliability of their detection results through advanced image processing algorithms [53].

Across a wide range of practical applications, infrared thermography NDT can not only detect internal defects in metallic and non-metallic materials; it can also measure the thickness of a wide range of materials, as well as the material and structural features beneath the surface of a test object [54,55,56,57,58,59,60,61,62,63]. With the rapid development of infrared scanners and artificial intelligence, non-destructive testing via infrared thermography does not need to be in direct contact with the object to be tested, which makes the testing process safer; thus, it is especially suitable for the inspection of bolts in high-temperature, high-pressure, or hazardous environments; for the inspection of bolts on high-temperature pipelines, for example, infrared thermography can be completed without making contact with the high-temperature surface. Furthermore, the significantly faster detection speeds than that of the traditional contact detection method quickly detect the fastening states and potential defects of bolts. At the same time, with high sensitivity and high resolution, this technique can detect small temperature differences; for example, by analyzing thermal changes during bolt loading, it can judge whether there are stress concentrations or cracks in the bolt [53]. However, its high accuracy and high resolution also impose greater requirements with respect to the professionalism of engineers and image processing technology, resulting in a greater need for professional personnel and software to operate in conjunction. At the same time, infrared thermography is mainly used to detect surface or near-surface defects; it struggles to accurately identify deep-seated defects such as deep cracks inside the bolts; in these cases, infrared thermography cannot provide sufficient detection accuracy.

### 3.5. Digital Image Inspection

**Figure 11:**
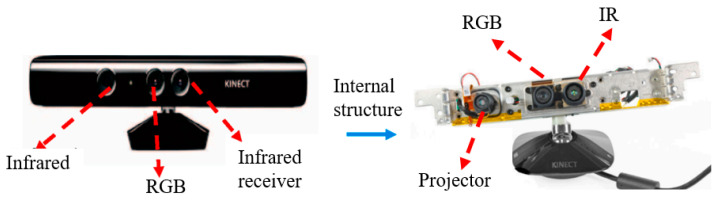
Structured light camera [60,61,62].

The ideal structural health inspection system for digital image inspection technology typically consists of two main components: (1) an in-built sensor network for collecting response measurements and (2) data analysis algorithms/software for interpreting the measurements in relation to the physical conditions of the structure (as shown in Figure 11) [64,65,66]. The objective of this technique is to convert the image information of the detected object into digital signals, which can then be analyzed and processed by image processing algorithms. The main steps include image acquisition, image pre-processing, feature extraction, and image recognition.

**Figure 12:**
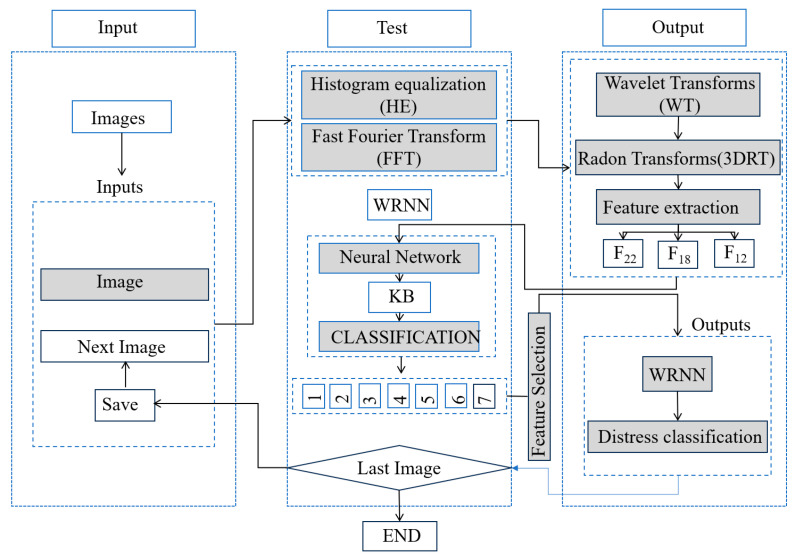
The detection process of digital image detection [64,67].

Image acquisition is the first step in digital image detection, and optical sensors (e.g., CCD or CMOS cameras) or X-ray detectors are usually used to obtain images of the inspected objects. The quality of image acquisition directly affects the subsequent processing, so it is necessary to select appropriate light sources, camera parameters, and imaging conditions. The purpose of image preprocessing is to remove noise and interference from the image and to enhance the useful information of the image. Common preprocessing methods include gray scale transformation, filter edge detection, and image segmentation. These methods can improve the contrast and clarity of the image and provide a better basis for subsequent feature extraction and image recognition. Feature extraction refers to the process of extracting useful information from an image that can be used to describe the various defects or features revealed. Common feature extraction methods include principal component analysis (PCA), independent component analysis (ICA), and pulse phasing. These methods can simplify complex image data into a small number of feature vectors for subsequent image recognition and analysis. Image recognition is the ultimate goal of digital image inspection, which achieves evaluation and diagnosis of the inspected object by identifying defects or features in the image. Common image recognition methods include template matching, neural network, support vector machine (SVM), and deep learning. These methods can automatically identify defects in images and provide accurate detection results. Figure 12 shows the detection process of digital detection techniques.

With the diversification of the complexity of table defects, digital image inspection techniques are also evolving. Park, J. H. et al. [68] proposed a light-invariant feature extraction algorithm, and the corrosion detection rate was increased to 92.5%. Yang, Z. et al. [69] developed a bolt missing identification model based on spatial location relationships, in which the false alarm rate was less than 3%. Zhang, X. et al. [70] fused thermograms and visible light images, achieving a loose bolt identification accuracy rate of 98.1% This shows that digital image inspection is a highly efficient tool for screening the surface defects of wind turbine tower bolts, but it needs to be employed alongside guided wave acoustic emission and other technologies to build a multi-level health monitoring system. It should be used in operation and maintenance (O&M) as a quick survey during downtime rather than as an alternative to online monitoring.

In addition, non-contact optical measurement technologies such as Digital Image Correlation (DIC) have gradually gained attention in the field of structural health monitoring. By analyzing the changes in speckle images on the surface of an object before and after deformation, DIC technology can achieve high-precision measurement of full-field strain and displacement, with advantages including non-contact, full-field measurement, and resistance to electromagnetic interference [71]. Studies have shown that DIC technology can be used for structural surface deformation monitoring and stress state evaluation [72]; for example, it has demonstrated application potential in the prestress estimation of membrane structures. Such methods provide a new technical approach for the indirect assessment of bolt connection status, and have unique value, especially in complex or harsh environments where it is difficult to arrange sensors.

### 3.6. Summary

Non-disassembly in situ inspection technology provides a key solution for the inspection and maintenance of wind turbine tower bolts. The four relatively established non-disassembly in situ online inspection technologies, based on different physical principles (acoustic, electromagnetic, optical, and thermal), for detecting defects in wind turbine tower bolts have been discussed above and found to be suitable for different application scenarios and material properties [73]. This section will summarize the strengths and weaknesses of the individual technologies and what types of defects they are suitable for. First of all, for ultrasonic detection, especially ultrasonic phased array-focused imaging technology, the advantages include fast detection speed, high sensitivity, real-time imaging, high-precision deep investigation of internal defects (≥0.5 mm cracks), and guided-wave detection for the long-distance detection of circumferential or axial defects, but the algorithms employed have various shortcomings [74]. Eddy-current detection is based on electromagnetic induction and is used to detect surface/near-surface cracks and corrosion. Its advantages include high detection speed and high sensitivity, but the disadvantages of this technology include its limitation to the detection of conductive materials, and it cannot produce images that communicate the depth of the defects. Ray detection can be used to visualize the internal structure of the bolt, including air holes and inclusions, etc. But due to the safety and protection requirements and high risk of radiation, the equipment is expensive and bulky, and is difficult to use for on-site monitoring in real time. Infrared thermal imaging detection via the analysis of temperature anomalies to locate defects has the advantages of being non-contact, applicable in high-risk environments (such as high-temperature bolts), and able to detect stress concentration, etc. However, the shortcomings of this technology include its inability to accurately and efficiently detect the depth of the defects, its high cost, and the need to rely on professional image processing and analysis of the collected data. Digital image inspection through image processing and AI algorithms to automatically identify defects relies on advanced algorithms to identify surface features. The process has a high degree of automation, but the technology is highly dependent on the image acquisition and post-processing capabilities, and the technology is more suited to surface-level defects [75].

Overall, this study presents several technologies for the non-disassembly in situ inspection of high-strength wind turbine bolt defects, taking into account the specific types of defects, the material characteristics of the bolts, the environmental conditions, and cost-effectiveness. We personally believe that the future of non-disassembly in situ bolt inspection lies in the combination of a variety of technologies. The integration of ultrasonic phased array depth capabilities, infrared thermal imaging, surface stress perception, and digital imaging via intelligent analysis could bring about the development of an intelligent, lightweight, and low-cost integrated system, employing AI to enhance the automatic identification of defects and improve accuracy assessment, which would more efficiently guarantee the structural safety of large-scale wind power facilities and their operational reliability. More detail is presented below.

## 4. Challenges and Developments

The proliferation of large-scale wind turbines poses three key challenges in bolt inspection: (1) dynamic wind load-induced signal drift, resulting in preload monitoring errors of up to 8% [76]; (2) elevated leakage rate of microcracks (<0.1 mm) in bolts of 10 MW or more [77]; and (3) inefficient high-altitude operation of 100 m scale towers, with manual inspection of a single bolt taking ≥15 min [78]. In order to solve the problems faced during the development of large-scale wind turbines, combining artificial intelligence with the above NDT technology is key, and deep learning algorithms enable stepwise accuracy enhancement in defect recognition via multi-source data fusion. There have already been applications of AI and deep learning to NDT technology, such as that of Zhang, J. et al., who found that the use of a convolutional neural network (CNN) to process the guided wave signals can classify 0.05 mm cracks (error < 5 μm [79]), and He, Y. et al. [80], who found that reinforcement learning optimized the drone inspection path to improve efficiency by 300%. Furthermore, to address the challenges of non-stationary signals and difficulties in feature extraction under dynamic wind load environments, advanced signal processing methods such as Instantaneous Spectral Entropy have been successfully applied to the condition monitoring of wind turbines, providing a novel technical approach for identifying early signs of bolt loosening from complex vibration or acoustic emission signals [81]. This method breaks through the traditional threshold limitation and exhibits cross-condition adaptation through feature self-extraction. In Zhang, H. et al. [82], migration learning adapts the laboratory model to the offshore salt spray environment, which significantly reduces the false alarm rate (<2%).

The in-depth integration of artificial intelligence (AI) and deep learning is becoming a core driving force to solve the above-mentioned problems. Traditional non-destructive testing methods rely heavily on experts’ experience to set thresholds, making it difficult to cope with complex and changing working conditions. In contrast, AI methods have achieved a leapfrog improvement in the accuracy and efficiency of defect identification through feature self-extraction and multi-source data fusion, while demonstrating strong adaptability across different working conditions.

Intelligent signal processing and defect identification: Deep learning-based methods can directly learn the deep features of defects from original or preprocessed detection signals (such as ultrasonic A-scan, guided waves, eddy current impedance diagrams, and thermal image sequences), enabling end-to-end automated defect classification and quantification. For example, researchers such as Zhang, J. (2023) [79] used a one-dimensional convolutional neural network (1D-CNN) to process guided wave signals, successfully classifying microcracks with a depth of only 0.05 mm, with a quantification error of less than 5 μm. Researchers such as Wang, T. (2021) [44] applied deep learning to eddy current array imaging, achieving automatic segmentation and mapping of corrosion areas with an accuracy of up to 98.2%; their subsequent research (2023) [82] successfully adapted the laboratory-trained model to the marine salt spray environment through transfer learning technology, significantly reducing the false alarm rate (<2%).

Intelligence and automation of the detection process: AI not only optimizes data analysis but is also reshaping the entire detection process. Researchers such as He, Y. (2022) [80] used a reinforcement learning (RL) algorithm to optimize the detection path of drones for wind turbine towers, increasing detection efficiency by 300%. Researchers such as Yang, Z. (2023) [69] developed a bolt missing identification model based on deep learning and spatial geometric relationships, realizing automatic analysis of drone images with a false alarm rate of less than 3%.

In order to solve the current key challenges faced in wind turbine bolt defect detection, the future construction of an AI-driven predictive maintenance system is very likely to become a major development trend, and it will likely be based on embedded MEMS sensor cluster real-time data acquisition, 5G transmission to the digital twin platform, and the use of a deep generative adversarial network (GAN) to simulate the crack expansion path [83], achieving the leap from “defect identification” to “life prediction”, and providing zero-downtime intelligent protection for 200 m+ ultra-high-altitude wind turbine bolts.

While the majority of existing research and technological advancements in non-destructive testing (NDT) for wind turbine bolts have focused on image-based data—such as those derived from ultrasonic phased array imaging, infrared thermography, and digital visual inspection—it is crucial to recognize that many NDT modalities inherently generate time-series or discrete data streams. These include ultrasonic A-scans, guided wave signals, acoustic emission bursts, eddy current impedance variations, and strain readings from fiber Bragg gratings. Such data types are particularly prevalent in real-time monitoring and continuous health assessment scenarios, where dynamic load variations and environmental fluctuations must be captured with high temporal resolution [84].

The analysis of time-series data presents unique challenges, including non-stationary signal behaviors, noise interference under operational conditions, and the need for sophisticated feature extraction techniques to identify early-stage defects. Traditional image-centric approaches may not fully leverage the temporal patterns embedded in these signals, which often contain critical information about defect initiation and progression.

Recent studies have begun to address this gap by integrating advanced signal processing and machine learning methods tailored for time-series analysis. For instance, techniques such as wavelet transforms, empirical mode decomposition, and recurrent neural networks (RNNs) have been employed to enhance the detection sensitivity and accuracy of bolt loosening and micro-crack identification under dynamic loading conditions [79,85]. Notably, a study leveraging “Instantaneous Spectral Entropy” has demonstrated robust performance in extracting defect-related features from acoustic emission signals in wind turbine structures, providing a promising direction for handling non-stationary signals in real-world environments [81].

Furthermore, the integration of multi-modal data fusion—combining image, time-series, and discrete sensor data—holds great potential for developing comprehensive digital twin models of wind turbine bolted connections. Such models can simulate crack propagation paths under varying operational stresses, enabling predictive maintenance strategies that go beyond mere defect identification [83,86].

Therefore, future research should prioritize the development of unified frameworks capable of processing both image and non-image NDT data, leveraging deep learning architectures such as 1D-CNNs for time-series and Transformers for sequential data, to achieve a more holistic and adaptive bolt health monitoring system. This approach is supported by recent advancements in AI-driven signal analysis, as highlighted in a comprehensive review on intelligent NDT methodologies [87].

## 5. Summary

In this article, the relevant NDT techniques for wind turbine bolt defects are reviewed. A variety of inspection techniques are discussed for two different scenarios, namely, disassembly and non-disassembly, where disassembly includes magnetic particle inspection, penetration inspection, and intelligent torque inspection, and non-disassembly includes ultrasonic inspection, guided wave inspection, eddy-current inspection, radiographic inspection, infrared thermographic inspection, fiber-optic grating sensing inspection, and digital image inspection. The principal advantages and disadvantages of the above disassembly and non-disassembly detection technologies are also introduced and discussed.

While the aforementioned non-destructive testing technologies each have their own advantages, their performance in actual wind farm environments varies significantly, especially between harsh marine environments and relatively stable onshore environments. Offshore wind turbine bolts are long-term exposed to highly corrosive environments with high humidity and heavy salt spray, and are more affected by wind and wave loads, which place more stringent requirements on testing technologies. For example, although eddy current testing is sensitive to surface cracks and has a fast detection speed, its signals are easily interfered with by salt scales and anti-corrosion coatings on the bolt surface, resulting in a decrease in signal-to-noise ratio and requiring frequent calibration. Although ultrasonic phased arrays can accurately image internal defects, the stability of the probe couplant and the precise positioning of the probe pose great challenges in high-altitude and windy operating environments. In contrast, guided wave testing shows unique advantages in the detection of hard-to-reach offshore bolts due to its ability to achieve long-distance scanning with a single-point excitation, but its wave propagation characteristics are easily affected by changes in ambient temperature, requiring supporting temperature compensation algorithms. Infrared thermographic testing, however, struggles to form reliable thermal contrast due to uneven heat dissipation caused by sea winds, resulting in low reliability in offshore applications.

Therefore, technology selection must be based on specific application scenarios: onshore wind farms can more flexibly adopt contact-based, high-precision disassembly or semi-disassembly methods (such as magnetic particle testing and ultrasonic testing); for offshore wind farms with short maintenance windows and harsh environments, priority should be given to the development of non-contact, highly automated non-disassembly technologies (such as guided wave testing and visual inspection carried by drones), and the promotion of multi-technology integration and artificial intelligence-assisted analysis to cope with testing challenges under complex working conditions.

The three types of disassembly detection technologies based on different physical principles (electromagnetism and optomechanics) are suitable for different application scenarios and material properties. With the characteristics of high sensitivity and intuition, they are widely used in industrial manufacturing, aerospace, railway transportation, etc. However, because of their inevitable need for the disassembly of the measured parts, they greatly increase the time and human cost, relying heavily on human analysis and evaluation, which is subject to human error, and their practical application faces multiple difficulties; therefore, further development is still required in order to improve the automation of disassembly testing technologies and to quantitative analyze their capabilities.

Non-disassembly inspection technology can detect defects without disassembling the bolt, thus significantly improving inspection efficiency and reducing equipment downtime. Among these technologies, ultrasonic testing (UT)—which uses ultrasonic wave propagation of the reflection and refraction characteristics of the material to detect bolt defects—has the most obvious advantages. It can detect micron-level defects and is suitable for detecting cracks, porosity inclusions, and other complex defects inside the bolt and at the connection between the head and the rod. Furthermore, these defects can be imaged in real time, visualizing their location and shape. This technology is suitable for a variety of materials and structures and is not subject to the interference from the bolt surface coating, i.e., the oxide layer, which is widely used.

In addition to ultrasonic inspection, other non-disassembly in situ inspection techniques, such as guided wave inspection, eddy current inspection, radiographic inspection, infrared thermal imaging, fiber-optic grating sensing inspection, and digital image inspection, are each suitable for different inspection needs. Guided wave inspection is suitable for long-distance inspection, eddy current inspection is suitable for surface and near-surface defects, radiographic inspection is suitable for internal defects in complex structures, infrared thermal imaging is suitable for surface defects, fiber-optic grating sensing is suitable for the real-time monitoring of stress distribution and fatigue damage in bolts, and digital image inspection is suitable for the initial screening of surface defects.

Although the advantages of non-disassembly, non-destructive testing technologies are obvious, the diversity and complexity of their defects still pose challenges, such as the time necessary to complete a large number of inspection tasks; the accurate quantitative analysis of the specific size, shape, location, and other characteristics of the defect; and the obtainment of a good environmental adaptability, along with various other requirements. Therefore, it is still necessary to further improve the automation, intelligence, and quantitative analysis capabilities of non-disassembly, non-destructive testing technologies through continuous innovation and development to provide a more reliable guarantee of the safety of wind turbine bolts. Examples may include the combination of artificial intelligence and machine learning algorithms to achieve automated defect identification and assessment, and to improve the detection efficiency and accuracy, as well as the use of ultrasonic phased array technology to obtain information on multiple ultrasonic echoes through the setup of different phased array elements, combined with a number of algorithms employed to achieve more complex imaging of bolt defects, and so on.

## Figures and Tables

**Table 1:** Types of wind turbine bolts and working conditions.

Bolt Type	Working Part	Role	Key Characteristics
High-strength bolts	Tower sections, nacelle to tower, generator to nacelle, blades to hub, and other critical parts	Withstand high tensile/shear loads to ensure structural rigidity; transmit dynamic loads, such as wind loads and gravity loads	High tensile strength, heat-treated, preload-sensitive
Stainless steel bolts	In wet environments or where corrosion resistance is required, e.g., tower connections in offshore wind farms, blade connections, etc.	Maintain connection integrity in corrosion-resistant environments; reduce corrosion failures caused by salt spray/humidity	316 L stainless steel, sacrificing strength for corrosion resistance
Anchor bolts	The foundation part of the wind turbine, used to securely attach the wind turbine structure to the ground	Transmits the overall overturning moment to the foundation; resists extreme wind loads, seismic loads, etc.	Large diameter, embedded design, grouting process relies on
Titanium bolts	Lightweight design and increased durability in areas such as rotor blade connections in wind turbines	Lightweight design, density of 4.5 g/cm^3^; high specific strength, strength/weight better than steel; seawater corrosion resistance	Anodized surface, high cost
Adjustable bolts	Wind turbine rotor blade connections and bearing connections that require automatic adjustment of slackness	Word adjustment compensation for dynamic loads; reduces the risk of vibration-induced loosening	Built-in disk, spring/hydraulic compensation mechanism, real-time monitoring

**Table 2:** Comparative analysis of non-destructive testing methods for high-strength bolt defects in wind turbines.

	Method	Principle	Characteristics	Shortcomings	Applicable Scenarios
Disassembly Inspection Technology	Magnetic Particle Inspection	When a ferromagnetic material is magnetized, a leakage field is formed if there are defects, such as cracks on or near the surface	High sensitivity (0.1 µm), fast detection of individual bolts, and low equipment costs	Difficult to determine the depth of defects (≤2 mm)	Detection of surface cracks and inclusions
	Penetration test	The penetrant is coated on the bolt surface to penetrate deep into the surface opening defects	Suitable for all bolt materials, economical, high sensitivity (0.4 µm)	Not possible to detect closed defects or near-surface (depth ≥ 5 µm) defects	Detects surface cracks, porosity, corrosion pits
	Intelligent torque detection	Using high-precision torque sensors or intelligent wrenches to measure the bolt’s torque value	Real-time monitoring, easy data recording and analysis	Requiring high accuracy of the equipment	Determining if the bolt meets the standard
Non-disassembly In Situ Inspection Technology	Ultrasonic inspection	Using ultrasonic interaction with defects to obtain information on defects in high-strength bolts, and then judging the presence or absence of defects	Non-destructive, high detection accuracy and high sensitivity; can detect the defects inside the bolt (≥0.5 mm deep cracks)	Requires a coupling agent, such as water or gel	Suitable for defects such as cracks, porosity, inclusions
	Guided wave detection	The use of guided waves to determine the presence or absence of defects	It saves testing costs and improves testing efficiency	Sensitive to the geometry of the bolt end	Sensitive to corrosion and cracks inside the bolt
	Eddy current detection	An alternating magnetic field induces eddy currents on the surface of conductive bolts, defects will disturb the distribution of eddy currents	Good detection of surface defects (aluminum < 3 mm); no need for coupling agent	Unable to detect internal or deep defects in the bolt	Suitable for surface and near-surface defect detection
	Radiographic inspection	The use of rays to penetrate the bolt and the detection of internal defects by imaging the difference	Intuitive operation; sensitive to the imaging of defects high resolution (resolution up to 10 lp/mm)	Bulky and costly equipment, not suitable for rapid on-site inspection	Sensitive to volumetric defect detection and imaging intuitive
	Infrared thermal imaging	Locate defective areas by actively heating the bolt or detecting its natural thermal field	Non-contact; can scan large areas quickly	Significantly affected by ambient temperature	Suitable for bolt surface and near-surface debonding
	Fiber optic grating sensing detection	Pre-embedded fiber optic grating on the surface or inside the bolt, indirectly inferring defects	High accuracy and sensitivity; real-time monitoring	Need to pre-embed or paste optical fiber	Real-time monitoring of the strain state of the bolt
	Digital image detection	Use of the high-resolution camera to capture the bolt surface image	Non-contact; high accuracy and resolution (spatial resolution of 0.01 mm)	Unable to detect defects inside the bolt	Suitable for initial screening of defects on the bolt surface

## Data Availability

The data that support the fundings of this study are available from the corresponding author upon reasonable request.
